# Murine Fecal Microbiota Transplantation Alleviates Intestinal and Systemic Immune Responses in *Campylobacter jejuni* Infected Mice Harboring a Human Gut Microbiota

**DOI:** 10.3389/fimmu.2019.02272

**Published:** 2019-09-24

**Authors:** Markus M. Heimesaat, Katharina Mrazek, Stefan Bereswill

**Affiliations:** Institute of Microbiology, Infectious Diseases and Immunology, Gastrointestinal Microbiology Research Group, Charité - University Medicine Berlin, Corporate Member of Freie Universität Berlin, Humboldt-Universität zu Berlin, Berlin Institute of Health, Berlin, Germany

**Keywords:** *Campylobacter jejuni* infection, fecal microbiota transplantation, anti-inflammatory intervention strategies, human microbiota associated mice, host-pathogen-interactions

## Abstract

Human campylobacteriosis constitutes a zoonotic food-borne disease and a progressively rising health burden of significant socioeconomic impact. We have recently shown that conventional mice are protected from *Campylobacter jejuni* infection, which was not the case for human microbiota associated (hma) mice indicating that the host-specific gut microbiota composition primarily determines susceptibility to or resistance against *C. jejuni* infection. In our present preclinical intervention study we addressed whether gut microbiota changes in stably *C. jejuni* infected hma mice following murine fecal microbiota transplantation (mFMT) could alleviate pathogen-induced immune responses. To accomplish this, secondary abiotic C57BL/6 mice were generated by broad-spectrum antibiotic treatment, perorally reassociated with a complex human gut microbiota and challenged with *C. jejuni* by gavage. Seven days later *C. jejuni* infected hma mice were subjected to peroral mFMT on 3 consecutive days. Within a week post-mFMT fecal pathogenic burdens had decreased by two orders of magnitude, whereas distinct changes in the gut microbiota composition with elevated numbers of lactobacilli and bifidobacteria could be assessed. In addition, mFMT resulted in less *C. jejuni* induced apoptotic responses in colonic epithelia, reduced numbers of macrophages and monocytes as well as of T lymphocytes in the large intestinal mucosa and lamina propria and in less distinct intestinal pro-inflammatory cytokine secretion as compared to mock challenge. Strikingly, inflammation dampening effects of mFMT were not restricted to the intestinal tract, but could also be observed systemically as indicated by elevated serum concentrations of pro-inflammatory cytokines such as TNF-α, IL-12p70, and IL-6 in *C. jejuni* infected hma mice of the mock, but not the mFMT cohort. In conclusion, our preclinical mFMT intervention study provides evidence that changes in the gut microbiota composition which might be achieved by pre- or probiotic formulations may effectively lower intestinal *C. jejuni* loads, dampen both, pathogen-induced intestinal and systemic inflammatory sequelae and may represent a useful tool to treat continuous shedding of *C. jejuni* by asymptomatic carriers which is critical in the context of food production, hospitalization and immunosuppression.

## Introduction

Human campylobacteriosis is among the four most prevalent global causes of diarrheal morbidities, whereas *Campylobacter jejuni* even constitutes the most common bacterial etiologic agents of human gastroenteritis with increasing prevalences worldwide (1, 2). In 2016, more than 250,000 cases of campylobacteriosis were reported in the European Union and the European Economic Area with an incidence of 66 cases per 100,000 subjects (3). One needs to take into consideration, however, that the number of unreported cases might be much higher due to asymptomatic carriers and to difficulties in detection given the fastidious growth requirements of the bacteria (4). The zoonotic pathogens are part of the commensal gut microbiota of warm-blooded wild and domestic animals. Humans become infected by consumption of undercooked contaminated meat derived from *Campylobacter* colonized livestock animals such as poultry, but also swine and cattle or by ingestion of *C. jejuni* containing surface waters (5, 6). Infected patients are either asymptomatic, present with rather mild symptoms or suffer from abdominal cramps, fever, watery or even inflammatory and bloody diarrhea (7, 8). Severely affected intestinal tissues are histologically characterized by elevated immune cell counts, crypt abscesses and ulcerations (9, 10). In most cases, symptoms are self-limiting, resolve within 1 week and require symptomatic therapy only (11). Antibiotic treatment with macrolides or fluoroquinolones, however, might be indicated in severe cases mostly affecting immunocompromised patients (7, 8, 11). In rare cases, post-infectious sequelae such as the Guillain-Barré syndrome, Miller Fisher syndrome, Reiter's syndrome, or reactive polyarthritis might arise (12–14). The exact mechanisms underlying *C. jejuni*-host interactions are yet only incompletely understood. One of the reasons for this dilemma is that appropriate *C. jejuni* infection and inflammation models have been missing for many years. Mice are convenient *in vivo* vertebrate model organisms, but display a strong physiological colonization resistance against *C. jejuni* infection when bred and maintained under standard specific pathogen free (SPF) conditions (15–17). Whereas, conventionally colonized wildtype mice were protected from pathogenic colonization following peroral *C. jejuni* challenge even of high loads, the pathogen could stably establish alongside the gastrointesinal tract of mice in which the gut microbiota had been depleted following broad-spectrum antibiotic treatment (15, 16). Reassociation of microbiota-depleted (i.e., secondary abiotic) mice with conventional murine gut microbiota via peroral fecal microbiota transplantation (FMT), however, could restore the colonization resistance against the pathogen, which was not the case when microbiota depleted mice were reassociated with fecal microbiota derived from human donors (15, 16). Stable intestinal *C. jejuni* colonization of microbiota depleted as well as of human gut microbiota associated (hma) mice was further associated with pronounced pro-inflammatory immune responses mimicking key features of human campylobacteriosis (15). In our present preclinical intervention study we therefore addressed whether gut microbiota changes in stably infected *C. jejuni* mice harboring a human gut microbiota by peroral FMT derived from murine donors could lower intestinal pathogenic loads and dampen induced pro-inflammatory immune responses.

## Materials and Methods

### Ethics Statement

After approval by the commission for animal experiments headed by the “Landesamt für Gesundheit und Soziales” (LaGeSo, Berlin, registration number G0097/12 and G0039/15) mouse experiments were conducted in accordance with the European Guidelines for animal welfare (2010/63/EU). Clinical conditions of mice were assessed once a day.

### Introduction of Human Gut Microbiota Into Murine Microbiota Depleted Mice

**Figure 1:**
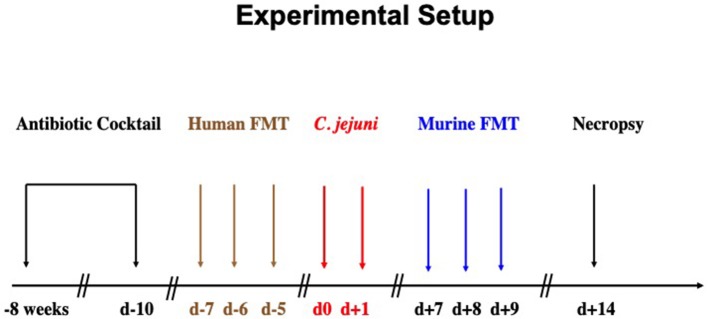
Experimental setup. Secondary abiotic mice were generated by broad-spectrum antibiotic treatment for 8 weeks. Three days before human fecal microbiota transplantation (FMT) the antibiotic cocktail was replaced by sterile tap water to guarantee antibiotic washout. Microbiota depleted mice were then subjected to human FMT on 3 consecutive days [i.e., day (d) −7, d−6, d−5]. To assure stable establishment of the human gut microbiota within the murine host, mice were kept for another 7 days before *C. jejuni* infection. On d0 and d+1, human microbiota associated (hma) mice were perorally subjected to *C. jejuni* by gavage. One week later, *C. jejuni* infected hma mice were treated with oral FMT derived from murine donors (d+7, d+8, d+9) and surveyed until necropsy on d+14.

C57BL/6j mice were reared under SPF conditions in the same unit of the Forschungseinrichtungen für Experimentelle Medizin (FEM, Charité - University Medicine Berlin). In order to override physiological colonization resistance and assure stable gastrointestinal *C. jejuni* colonization upon peroral challenge lateron (15), the murine gut microbiota was depleted by broad-spectrum antibiotic treatment as stated earlier (15, 18, 19). In brief, at the age of 6–8 weeks mice were treated with ampicillin plus sulbactam (1 g/L; Ratiopharm, Germany), vancomycin (500 mg/L; Cell Pharm, Germany), ciprofloxacin (200 mg/L; Bayer Vital, Germany), imipenem (250 mg/L; MSD, Germany), and metronidazole (1 g/L; Fresenius, Germany) via autoclaved drinking water for 8 weeks (*ad libitum*). Three days before human fecal microbiota transplantation, the antibiotic cocktail was replaced by autoclaved tap water (Figure 1).

To introduce a complex gut microbiota of human origin into the murine host, microbiota-depleted mice were subjected to peroral human FMT on 3 consecutive days as described earlier (20, 21). In brief, fresh fecal samples free of enteropathogenic bacteria, viruses and parasites were collected from five individual healthy human donors, dissolved in sterile phosphate buffered saline (PBS, Gibco, Life Technologies, UK), aliquoted and stored at −80°C as state elsewhere (15, 22–24). Immediately before FMT, individual fecal aliquots were thawed, pooled and applied to mice perorally by gavage in a total volume of 0.3 mL (15, 22–24). To assure proper establishment of the complex human microbiota in the murine host, mice were kept for 7 days after the initial human FMT until *C. jejuni* infection (Figure 1).

### *C. jejuni* Infection and Fecal Loads

For infection, a stock solution of *C. jejuni* 81–176 strain that had been stored at −80°C was thawed, aliquots streaked onto karmali agar (Oxoid, Wesel, Germany) and incubated in a microaerophilic atmosphere at 37°C for 48 h. Immediately before peroral infection of mice, bacteria were harvested in sterile PBS to a final inoculum of 10^9^ bacterial cells.

Female and male hma mice (4 months of age) were perorally infected by gavage (in a total volume of 0.3 mL PBS) on 2 consecutive days starting on day (d) 0 and d1 (Figure 1). *C. jejuni* loads were monitored in fecal samples over time post-infection as reported previously (15, 25). In brief, serial dilutions of fecal samples were dissolved in sterile PBS, streaked onto karmali agar and quantitatively assessed 48 h following incubation in a microaerophilic atmosphere at 37°C. The detection limit of viable pathogens was ≈100 CFU per g.

### Murine Fecal Microbiota Transplantation

At days 7, 8, and 9 post-infection, *C. jejuni* infected hma mice were treated with FMT from murine donors (Figure 1) as described earlier (15, 22–24, 26). In brief, fresh murine fecal samples were collected from 10 age and sex matched conventionally colonized (i.e., SPF) mice, pooled, dissolved in 10 mL sterile PBS and the supernatant used as murine donor suspension. Sex and age matched *C. jejuni* infected hma mice were either perorally treated with 0.3 mL of murine donor suspension by gavage or received PBS only as mock control animals (15, 22–24, 26). Immediately before either FMT, aliquots from both, human and murine donor solutions were collected for quantitative molecular analyses of main intestinal bacterial communities as described elsewhere (15, 18, 27).

### Gut Microbiota Composition

DNA was extracted from fecal samples or fecal donor suspensions as stated earlier (18, 28). In brief, DNA was quantified by using Quant-iT PicoGreen reagent (Invitrogen, UK) and adjusted to 1 ng per μL. Then, total eubacterial loads as well as the main bacterial groups abundant in the murine and human intestinal microbiota including enterobacteria, enterococci, lactobacilli, bifidobacteria, *Bacteroides/Prevotella* species, *Clostridium coccoides* group, *Clostridium leptum* group, and *Mouse Intestinal Bacteroides* were determined by quantitative real-time polymerase chain reaction (qRT-PCR) with species-, genera- or group-specific 16S rRNA gene primers (Tib MolBiol, Germany) as described previously (15, 27, 29) and numbers of 16S rRNA gene numbers per ng DNA of each sample assessed.

### Sampling Procedures

At day 14 post-infection, mice were sacrificed (Figure 1) by isofluran inhalation (Abbott, Germany). *Ex vivo* biopsies from colon and mesenteric lymph nodes (MLN) were taken under sterile conditions. Large intestinal samples were collected from each mouse in parallel for microbiological, immunohistopathological, and immunological analyses.

### Immunohistochemistry

Colonic *ex vivo* biopsies were immediately fixed in 5% formalin, embedded in paraffin, and subjected to *in situ* immunohistochemical analyses as reported previously (27, 30–32). In brief, for detection of apoptotic epithelial cells, macrophages/monocytes and T lymphocytes paraffin sections (5 μm) were stained with primary antibodies directed against cleaved caspase 3 (Asp175, Cell Signaling, Beverly, MA, USA, 1:200), F4/80 (# 14-4801, clone BM8, eBioscience, San Diego, CA, USA, 1:50), and CD3 (#N1580, Dako, 1:10), respectively. Then, positively-stained cells were quantitatively examined by a blinded independent investigator (light microscopy, magnification 100x and 400x). The average number of respective positively-stained cells for each individual section was determined within at least six high power fields (HPF, 0.287 mm^2^, 400x magnification).

### Pro-inflammatory Cytokine Secretion

Large intestinal *ex vivo* biopsies were cut longitudinally and washed in PBS. MLN (3 lymph nodes) or strips of approximately 1 cm^2^ colonic tissues were placed in 24-flat-bottom well culture plates (Nunc, Germany) with 500 μL serum-free RPMI 1640 medium (Gibco, life technologies, UK) and supplemented with penicillin (100 U/mL) and streptomycin (100 μg/mL; PAA Laboratories, Germany). After 18 h at 37°C, culture supernatants and serum samples were tested for TNF-α, IFN-γ, IL-6, and IL-12p70 by the Mouse Inflammation Cytometric Bead Assay (CBA; BD Biosciences, Germany) on a BD FACSCanto II flow cytometer (BD Biosciences).

### Statistical Analysis

Medians and levels of significance were determined using Mann-Whitney test (GraphPad Prism v7, USA) for pairwise comparisons of not normally distributed data and using the one-sided ANOVA with Tukey post-correction or the Kruskal-Wallis test with Dunn's post-correction for multiple comparisons as indicated. Two-sided probability *p* ≤ 0.05 were considered significant. Experiments were reproduced three times.

## Results

### Lower Intestinal Pathogenic Burdens Following Murine Fecal Microbiota Transplantation of *C. jejuni* Infected Mice Harboring a Human Gut Microbiota

**Figure 2:**
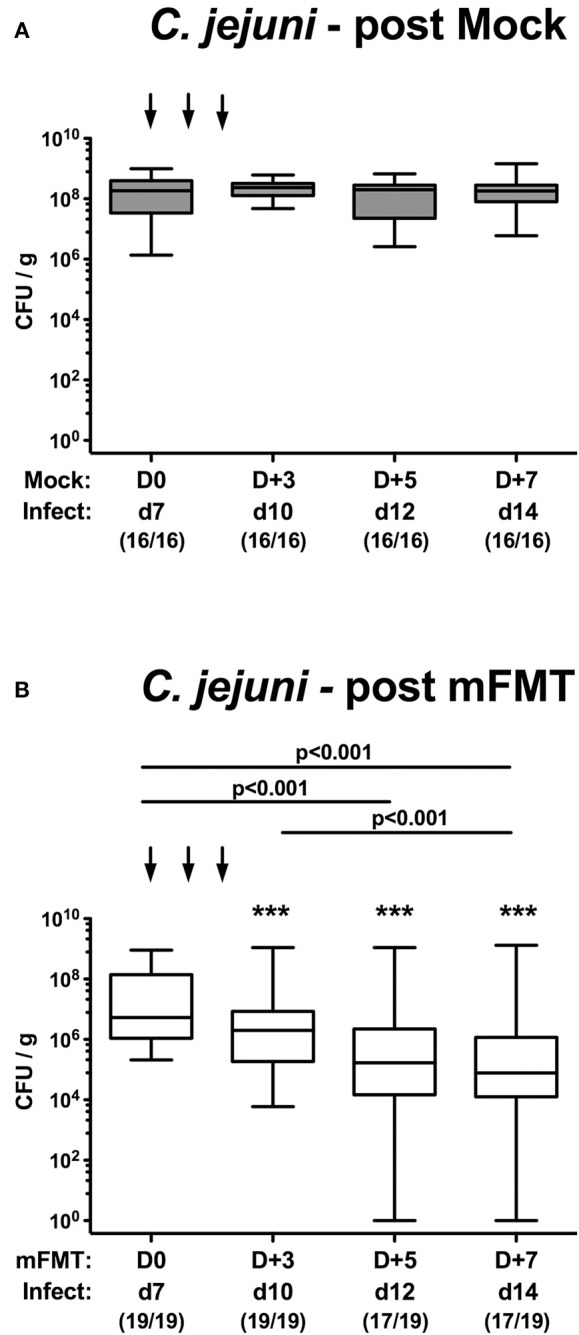
Kinetic survey of fecal *C. jejuni* shedding following murine fecal microbiota transplantation of infected mice harboring a human gut microbiota. Mice with a human gut microbiota were perorally infected with *C. jejuni* on day (d) 0 and d1 (Infect) and subjected to **(A)** vehicle (mock) treatment or to **(B)** murine fecal microbiota transplantation (mFMT) on d7, d8, and d9 post-infection (arrows; D0, D+1, D+2). Immediately before and after mFMT fecal samples were taken at defined time points as indicated to assess intestinal pathogenic loads by culture (expressed as colony forming units per gram, CFU/g). Box plots represent the 75th and 25th percentiles of medians (black bar inside the boxes). The total range, significance levels (*p*-values) determined by the Kruskal-Wallis test and Dunn's post-correction and the numbers of *C. jejuni* positive mice out of the total number of animals (in parentheses) are given. Data were pooled from four independent experiments. ^***^p < 0.001 comparing bacterial loads in mock vs. mFMT treated mice at identical time points (Mann Whitney *U*-test).

Mice with a human gut microbiota were perorally infected with *C. jejuni* on days 0 and 1 and subjected to murine fecal microbiota transplantation (mFMT) or received vehicle only on 3 consecutive days starting at day 7 post-infection (Figure 1). Immediately before and after the mFMT we surveyed the intestinal pathogenic loads over time by cultural analyses of fecal samples. Following peroral infection, mice from the mock control group harbored more than 10^8^ *C. jejuni* cells until the end of the observation period (Figure 2A). As early as 72 h following the latest mFMT (D+5), however, fecal *C. jejuni* burdens had declined (*p* < 0.001; Figure 2B), whereas 1 week following the initial mFMT (D+7), the median fecal pathogen loads were approximately two orders of magnitude lower as compared to those obtained before the intervention (*p* < 0.001; Figure 2B). Notably, single animals had even completely lost the pathogen from their intestines as early as 5 days following the initial mFMT (D+5; Figure 2B). Furthermore, in mice from the mFMT cohort fecal *C. jejuni* loads were lower at individual time points post- intervention as compared to mock counterparts (*p* < 0.001; Figure 2). Hence, mFMT could sufficiently lower pathogenic burdens in the intestines of hma mice.

### Gut Microbiota Changes Following Murine Fecal Microbiota Transplantation of *C. jejuni* Infected Mice Harboring a Human Gut Microbiota

**Figure 3:**
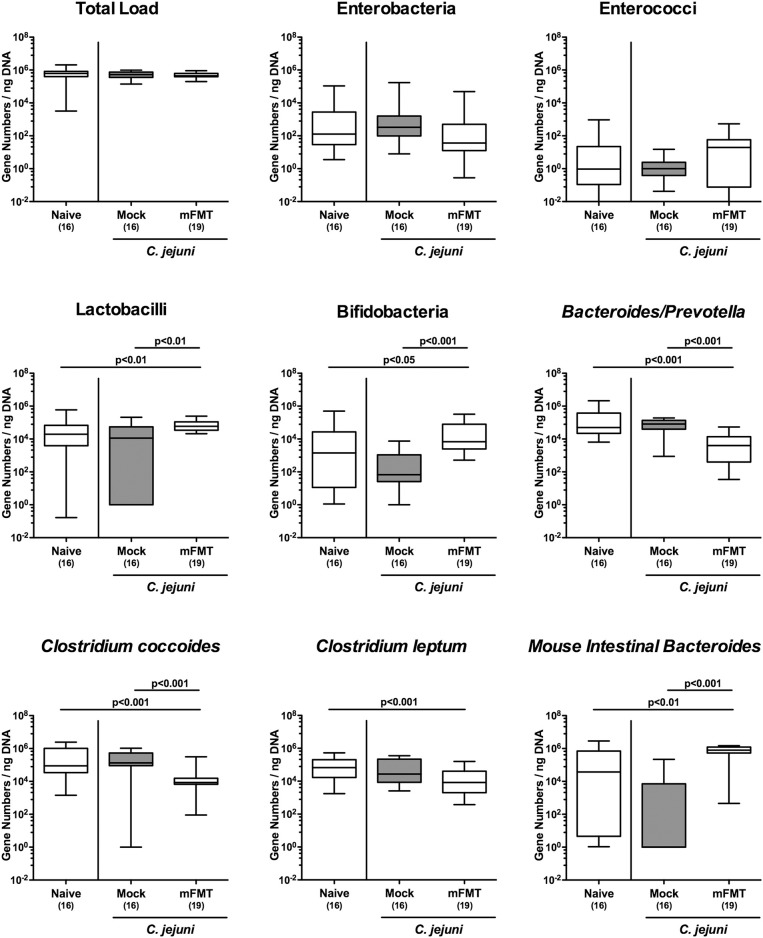
Changes in intestinal microbiota composition following murine fecal microbiota transplantation of *C. jejuni* infected mice harboring a human gut microbiota. Mice with a human gut microbiota were perorally infected with *C. jejuni* on day (d) 0 and d1 and subjected to murine fecal microbiota transplantation (mFMT) or to mock treatment on d7, d8, and d9 post-infection (i.e., D0, D+1, D+2) or received vehicle (mock). Seven days later (i.e., D+7), the fecal commensal microbiota composition was assessed applying culture-independent 16S rRNA methods quantitating the total eubacterial load and main bacterial groups as indicated (expressed as gene numbers per ng DNA). Box plots represent the 75th and 25th percentiles of medians (black bar inside the boxes). The total range and significance levels (*p*-values) determined by the Kruskal-Wallis test and Dunn's post-correction and numbers of analyzed animals (in parentheses) are indicated. Data were pooled from four independent experiments.

We further addressed to what extent the commensal gut microbiota composition of mice harboring a human gut microbiota had changed upon the mFMT applying culture-independent molecular methods. Seven days after the start of the intervention (D+7), mice from the mFMT cohort displayed higher numbers of lactobacilli (*p* < 0.01), bifidobacteria (*p* < 0.05–0.001), and *Mouse Intestinal Bacteroides* (*p* < 0.01–0.001) as compared to mice from the mock group and naive control animals, whereas fecal loads of *Bacteroides/Prevotella* species (*p* < 0.001), and *Clostridium coccoides* (*p* < 0.001) were lower in the former as compared to the latter (Figure 3). Furthermore, mFMT treated mice displayed lower fecal *Clostridium leptum* loads as compared to naive mice (*p* < 0.001; Figure 3). As expected, marked quantitative differences in the main commensal bacterial group abundant in the intestinal tract could be assessed when comparing the fecal solutions from human vs. murine donors (Figure S1). Hence, within 1 week mFMT results in distinct changes of the intestinal microbiota composition of hma mice.

### Less Pronounced Intestinal Apoptotic Cell and Immune Cell Responses Following Murine Fecal Microbiota Transplantation of *C. jejuni* Infected Mice Harboring a Human Gut Microbiota

**Figure 4:**
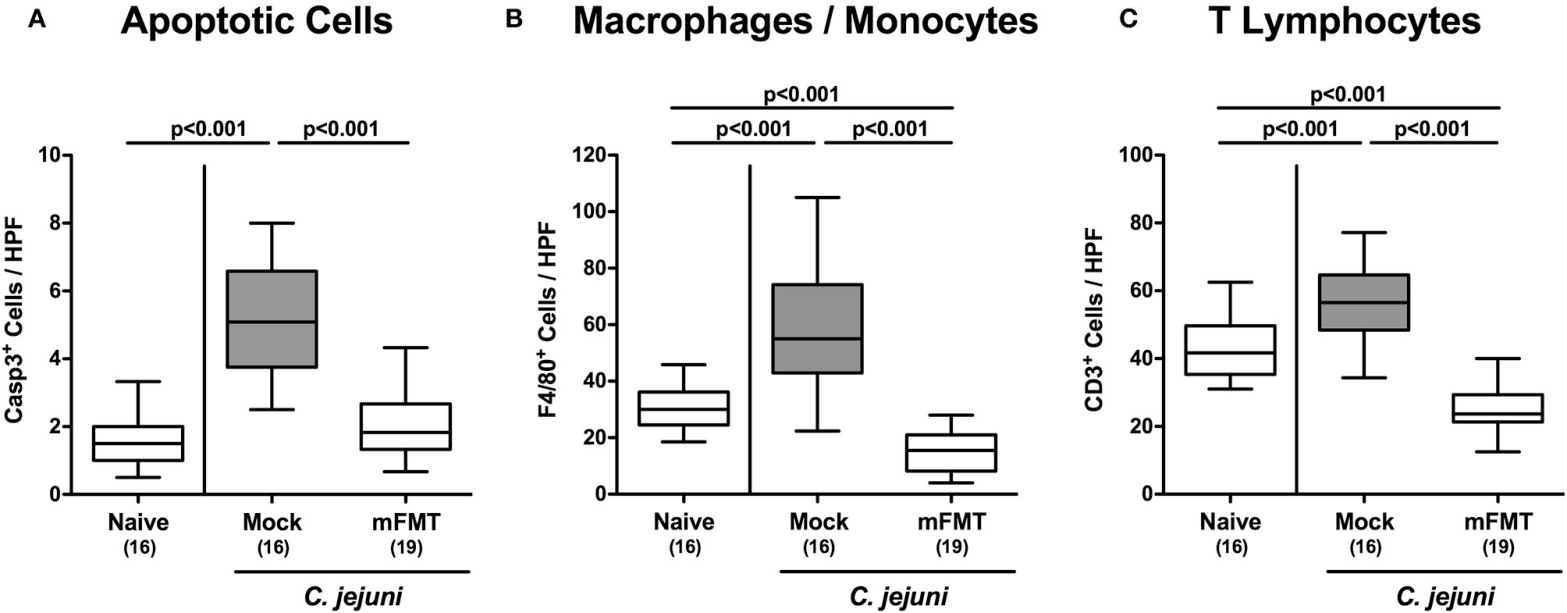
Colonic apoptotic epithelial and immune cell responses following murine fecal microbiota transplantation of *C. jejuni* infected mice harboring a human gut microbiota. Mice with a human gut microbiota were perorally infected with *C. jejuni* on day (d) 0 and d1 and subjected to murine fecal microbiota transplantation (mFMT) on d7, d8, and d9 post-infection (p.i.) or received vehicle (mock). On day 14 p.i., the average numbers of **(A)** caspase3+ (Casp3+) apoptotic epithelial cells, **(B)** F4/80+ macrophages and monocytes and of **(C)** CD3+ T lymphocytes were assessed from six high power fields (HPF, 400x magnification) per mouse in immunohistochemically stained colonic paraffin sections. Naive mice with a human gut microbiota served as uninfected and untreated controls. Box plots represent the 75th and 25th percentiles of medians (black bar inside the boxes). The total range, significance levels (*p*-values) determined by the one-sided ANOVA test with Tukey post-correction and the numbers of *C. jejuni* positive mice out of the total number of animals (in parentheses) are indicated. Data were pooled from four independent experiments.

We next addressed whether mFMT resulted in less pronounced *C. jejuni* induced inflammatory responses in the intestinal tract. *C. jejuni* infection was associated with multifold increased numbers of colonic apoptotic epithelial cells (*p* < 0.001), unless infected hma mice had been subjected to mFMT (n.s. vs. naive; Figure 4A; Figure S2A). At day 14 post-infection, mock control mice exhibited increased numbers of innate immune cell subsets such as macrophages and monocytes (*p* < 0.001; Figure 4B; Figure S2B) as well as of adaptive immune cells including T lymphocytes (*p* < 0.001; Figure 4C; Figure S2C) in their colonic mucosa and lamina propria, whereas respective immune cell populations were lower in infected mice after mFMT vs. mock application (*p* < 0.001; Figures 4B,C; Figures S2B,C). Hence, mFMT dampens *C. jejuni* induced apoptotic cell and immune cell responses in the large intestines of mice with a human gut microbiota.

### Less Intestinal Pro-inflammatory Cytokine Secretion Following Murine Fecal Microbiota Transplantation of *C. jejuni* Infected Mice Harboring a Human Gut Microbiota

**Figure 5:**
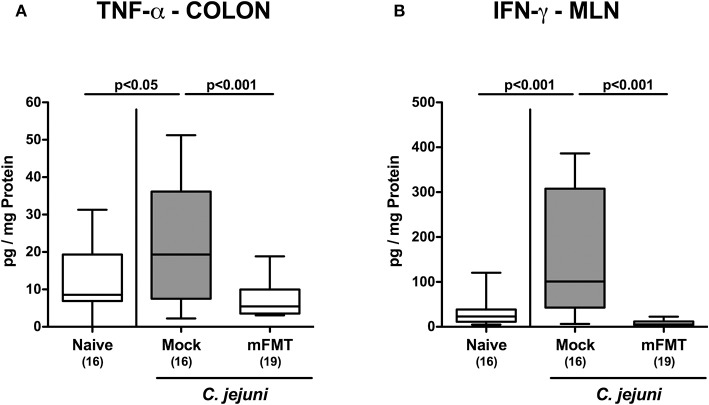
Intestinal pro-inflammatory cytokine secretion following murine fecal microbiota transplantation of *C. jejuni* infected mice harboring a human gut microbiota. Mice with a human gut microbiota were perorally infected with *C. jejuni* on day (d) 0 and d1 and subjected to murine fecal microbiota transplantation (mFMT) on d7, d8, and d9 post-infection (p.i.) or received vehicle (mock). On day 14 p.i., **(A)** TNF-α and **(B)** IFN-γ concentrations were measured in *ex vivo* biopsies derived from the colon and mesenteric lymph nodes (MLN), respectively. Naive mice with a human gut microbiota served as uninfected and untreated controls. Box plots represent the 75th and 25th percentiles of medians (black bar inside the boxes). The total range, significance levels (*p*-values) determined by the one-sided ANOVA test with Tukey post-correction and the numbers of *C. jejuni* positive mice out of the total number of animals (in parentheses) are indicated. Data were pooled from four independent experiments.

We further addressed whether the dampened *C. jejuni* induced immune responses upon mFMT were associated with less pro-inflammatory cytokine secretion in the intestinal tract. In fact, elevated TNF-α concentrations could be measured in colonic *ex vivo* biopsies taken from mock controls at day 14 post-infection (*p* < 0.05 vs. naive), which was, however, not the case when mice had been challenged with mFMT (*p* < 0.001 vs. mock; Figure 5A). In support, multifold increased IFN-γ concentrations were determined in MLN when taken from mock animals (*p* < 0.001 vs. naive), but not from mice following mFMT at day 14 post-infection (*p* < 0.001 vs. mock; Figure 5B). Hence, mFMT resulted in less distinct pathogen induced pro-inflammatory cytokine secretion in the intestinal tract in *C. jejuni* mice with a human gut microbiota.

### Less Systemic Pro-inflammatory Cytokine Secretion Following Murine Fecal Microbiota Transplantation of *C. jejuni* Infected Mice Harboring a Human Gut Microbiota

**Figure 6:**
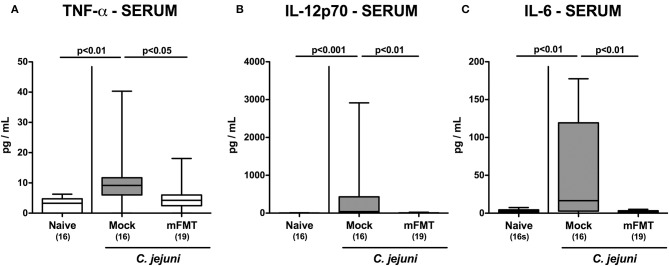
Systemic pro-inflammatory cytokine secretion following murine fecal microbiota transplantation of *C. jejuni* infected mice harboring a human gut microbiota. Mice with a human gut microbiota were perorally infected with *C. jejuni* on day (d) 0 and d1 and subjected to murine fecal microbiota transplantation (mFMT) on d7, d8, and d9 post-infection (p.i.) or received vehicle (mock). On day 14 p.i., **(A)** TNF-α, **(B)** IL-12p70, and **(C)** IL-6 concentrations were measured in serum samples. Naive mice with a human gut microbiota served as uninfected and untreated controls. Box plots represent the 75th and 25th percentiles of medians (black bar inside the boxes). The total range, significance levels (*p*-values) determined by the one-sided ANOVA test with Tukey post-correction or by the Kruskal-Wallis test and Dunn's post-correction and the numbers of *C. jejuni* positive mice out of the total number of animals (in parentheses) are indicated. Data were pooled from four independent experiments.

We further addressed whether the anti-inflammatory effects of mFMT in *C. jejuni* infected mice was restricted to the intestinal tract or also effective in the systemic compartment. Remarkably, serum concentrations of TNF-α, IL-12p70, and IL-6 were all elevated at day 14 following *C. jejuni* infection of mice in the mock cohort (*p* < 0.01–0.001 vs. naive; Figure 6), but not in the mFMT intervention cohort (*p* < 0.05–0.01 vs. mock; Figure 6). Hence, the anti-inflammatory properties of mFMT in *C. jejuni* infected mice were not restricted to the intestinal tract, but were also effective systemically.

## Discussion

The specific gut microbiota composition of the vertebrate host primarily determines susceptibity to or resistance against infections with enteropathogens including *C. jejuni* (15–17). The FMT as therapeutic application dates back to the Chinese Dong-jin dynasty in the fourth century and has very recently experienced a renaissance for the treatment of recurrent and refractory infections with enterotoxin-producing *Clostridioides difficile* strains, of inflammatory bowel diseases and of the chronic fatigue syndrome, for instance (33–39). Furthermore, FMT constitutes a promising antibiotics-independent therapeutic approach to combat carriage with (opportunistic) pathogens, particularly with multi-drug resistant strains. In support, we were able to show very recently that mFMT in hma mice on 3 consecutive days could effectively reduce intestinal burdens of multi-drug resistant *Pseudomonas aeruginosa* by almost four orders of magnitude, whereas individual mice had even completely lost the opportunistic pathogen (40).

Given that conventionally colonized mice as well as microbiota depleted mice that had been reassociated with a complex murine gut microbiota were protected from even high-dose *C. jejuni* infection as opposed to hma counterparts (15), we here evaluated mFMT as a potential intervention strategy to combat *C. jejuni* infection and pathogen-induced inflammatory sequelae. In order to mimick human gut microbiota conditions we had subjected microbiota depleted mice to peroral FMT from human donors before *C. jejuni* infection. As any other experimental model also the here applied hma mouse model has its limitations. We cannot exclude, for instance, that some members of the human fecal donor samples, in particular obligate anaerobic and other fastidious bacterial species, might be reduced during asservation and processing including freezing, thawing and FMT and/or did not fully establish within the gastrointestinal ecosystem of the murine host (20, 24). Moreover, several host-related and environmental factors might further affect the fate of the human fecal transplant over time such as the genetic background, the anatomical and mucosal immunological repertoire within the respective gastrointestinal compartment, the intraluminal milieu, as well as the housing conditions including diet of the challenged mice (24, 41–43). Under consideration of both, the limitations and the strengths of the applied experimental model, hma mice constitute worthwhile measures to reliably unravel the interactions between pathogens, gut commensals and host immune responses in health and disease. In fact, with respect to their microbiota “humanized” mice have been successfully used as tools to investigate the interactions between the vertebrate host and enteropathogens including *C. difficile, Salmonella*, and *C. jejuni* (15, 44, 45).

In our actual preclinical intervention study applying *C. jejuni* infected hma mice we were able to show that mFMT (i) resulted in changes of the gut microbiota composition, (ii) could lower the intestinal pathogen burdens by two log orders of magnitude, (iii) dampen *C. jejuni* induced apoptotic cell and immune cell responses in the large intestine that were associated with (iv) less distinct pro-inflammatory cytokine secretion in both, the large intestines and MLN. Strikingly, (v) the anti-inflammatory properties of mFMT were not restricted to the intestinal tract, but could also be observed systemically.

Our molecular survey of the main abundant intestinal bacterial groups and species revealed that the mFMT on 3 consecutive days did in fact result in changes of the gut microbiota composition of hma mice within 1 week post-intervention. In line with the observed quantitative differences of bacterial taxa from fecal suspensions derived from human vs. murine donors, the fecal microbiota at day 7 post-mFMT of hma mice was characterized by higher numbers of lactobacilli, bifidobacteria and *Mouse Intestinal Bacteroides*, whereas *Bacteroides/Prevotella* species and clostridia were lower as compared to pre-mFMT conditions. Comparable gut microbiota shifts could be observed following mFMT of hma mice that were carrying multi-drug resistant *Pseudomonas aeruginosa* in their intestines (40). It is tempting to speculate that (yet to be identified) specific “health-beneficial,” immune-modulatory bacterial commensal species within the complex gut luminal ecosystem might make the difference and provide anti-inflammatory properties following infection with enteropathogens including *C. jejuni*. Lactobacilli and bifidobacteria may be potential candidates since they are well known for their probiotic effects due to production of bacteriocins and short chain fatty acids subsequently creating hostile conditions for invading pathogens (46, 47). In support, we were able to show recently that peroral application of a single *Lactobacillus johnsonii* strain that had been isolated from a fecal sample taken from a healthy C57BL/6 mouse could effectively alleviate intestinal, extra-intestinal and, remarkably, even systemic pro-inflammatory immune responses upon *C. jejuni* infection of secondary abiotic mice (48). Additional studies further revealed potent anti-inflammatory effects of *L. johnsonii* in enteric including infectious diseases (49) mounting in commercial probiotic application (e.g., Nestlé LC1).

Bifidobacteria are considered key players in maintaining intestinal homeostasis. In fact, a delay in intestinal colonization with bifidobacteria rendered individuals more susceptible for morbidities during infancy and later in life (50). Several intestinal immunopathological conditions including inflammatory bowel diseases, celiac disease and irritable bowel syndrome have been associated with a perturbed gut microbiota (i.e., dysbiosis) with decreased or even absent intestinal bifidobacteria (51). Due to their anti-inflammatory properties defined bifidobacteria as well as lactobacilli strains have been introduced into commercial probiotic formulations such as VSL#3 and have been shown to effectively alleviate clinical symptoms and maintain remission in inflammatory bowel diseases (51). Furthermore, and supporting our results obtained from our actual mFMT intervention study, we demonstrated recently that VSL#3 application did not only dampen pro-inflammatory immune responses in the intestinal tract, but also in extra-intestinal and even systemic compartments upon *C. jejuni* infection of microbiota depleted mice (52).

It is thus highly likely that synergistic effects between different commensal bacterial species are more sufficient to exert potent anti-inflammatory in the combat of enteropathogenic including *C. jejuni* infections than single strains alone. In support, we were able to show previously that mFMT in microbiota depleted mice could induce more prominent anti-inflammatory responses than reassociation with a commensal murine *L. johnsonii* strain alone as indicated by more pronounced anti-inflammatory CD25 expression in intestinal as well as systemic immunological compartments following the former as compared to the latter intervention (26). It would therefore be utmost appreciable to characterize the gut luminal milieu in more detail in order to define distinct bacterial strains and/or metabolites that might be promising candidate molecules for future prophylactic or therapeutic application in humans and food animals.

In conclusion, our preclinical mFMT intervention study provides evidence that changes in the gut microbiota composition which might be achieved by pre- or probiotic formulations may effectively lower intestinal *C. jejuni* loads and dampen both, pathogen-induced intestinal and systemic inflammatory sequelae. Furthermore, the applied infection model provides a valuable tool to identify luminal intestinal molecules mediating colonization resistance for future treatment and prevention of *C. jejuni* infection and colonization in the vertebrate host and may represent a promising option to treat continuous shedding of *C. jejuni* by asymptomatic carriers which is critical in the context of food production, hospitalization, and immunosuppression.

## Data Availability Statement

All datasets generated for this study are included in the manuscript/Supplementary Files.

## Ethics Statement

The animal study was reviewed and approved by Landesamt für Gesundheit und Soziales (LaGeSo, Berlin).

## Author Contributions

MH designed and performed experiments, analyzed data, and wrote paper. KM performed experiments, analyzed data, and co-edited paper. SB provided advice in experimental design, critically discussed results, and co-edited paper.

### Conflict of Interest

The authors declare that the research was conducted in the absence of any commercial or financial relationships that could be construed as a potential conflict of interest.

## Supplementary Material

Figure S1 Microbiota composition of suspensions derived from human and murine donors used for fecal microbiota transplantation. Immediately before fecal microbiota transplantation from human and murine donors, the commensal microbiota composition was assessed in respective fecal suspensions (*n* = 3 each) applying culture-independent 16S rRNA methods quantitating the total eubacterial load (TL) and main bacterial groups including enterobacteria (EB), enterococci (EC), lactobacilli (LB), bifidobacteria (BB), *Bacteroides/Prevotella* species (BP), *Clostridium coccoides* group (CC), *Clostridium leptum* group (CL), and *Mouse Intestinal Bacteroides* (MIB) and expressed as gene numbers per ng DNA. Box plots represent the 75th and 25th percentiles of medians (black bar inside the boxes) and the total range is indicated. Data were pooled from three independent suspensions.

Figure S2 Representative photomicrographs illustrating colonic apoptotic epithelial and immune cell responses following murine fecal microbiota transplantation in *C. jejuni* infected mice harboring a human gut microbiota. Mice with a human gut microbiota were perorally infected with *C. jejuni* on day (d) 0 and d1 and subjected to murine fecal microbiota transplantation (mFMT) on d7, d8, and d9 post-infection (p.i.) or remained untreated (mock). Photomicrographs representative for four independent experiments illustrate the average numbers of **(A)** apoptotic epithelial cells (caspase3+), **(B)** macrophages and monocytes (F4/80+), and **(C)** T lymphocytes (CD3+), in at least six high power fields (HPF) as quantitatively assessed in colonic paraffin sections applying *in situ* immunohistochemistry at day 14 p.i. (100x magnification, scale bar 100 μm).

## Abbreviations

CFU: colony forming units
D, d: day
FMT: fecal microbiota transplantation
Hma: human microbiota associated
HPF: high power field
IFN: interferon
IL: interleukin
mFMT: murine fecal microbiota transplantation
MLN: mesenteric lymph nodes
n.s: not significant
PBS: phosphate buffered saline
qRT-PCR: quantitative real-time polymerase chain reaction
SPF: specific pathogen free
TNF: tumor necrosis factor.
