# Supplementary figures and images for: Murine Fecal Microbiota Transplantation Alleviates Intestinal and Systemic Immune Responses in Campylobacter jejuni Infected Mice Harboring a Human Gut Microbiota
Source: Front Immunol. 2019 Sep 24;10:2272. doi: 10.3389/fimmu.2019.02272 (PMC6768980; doi:10.3389/fimmu.2019.02272)

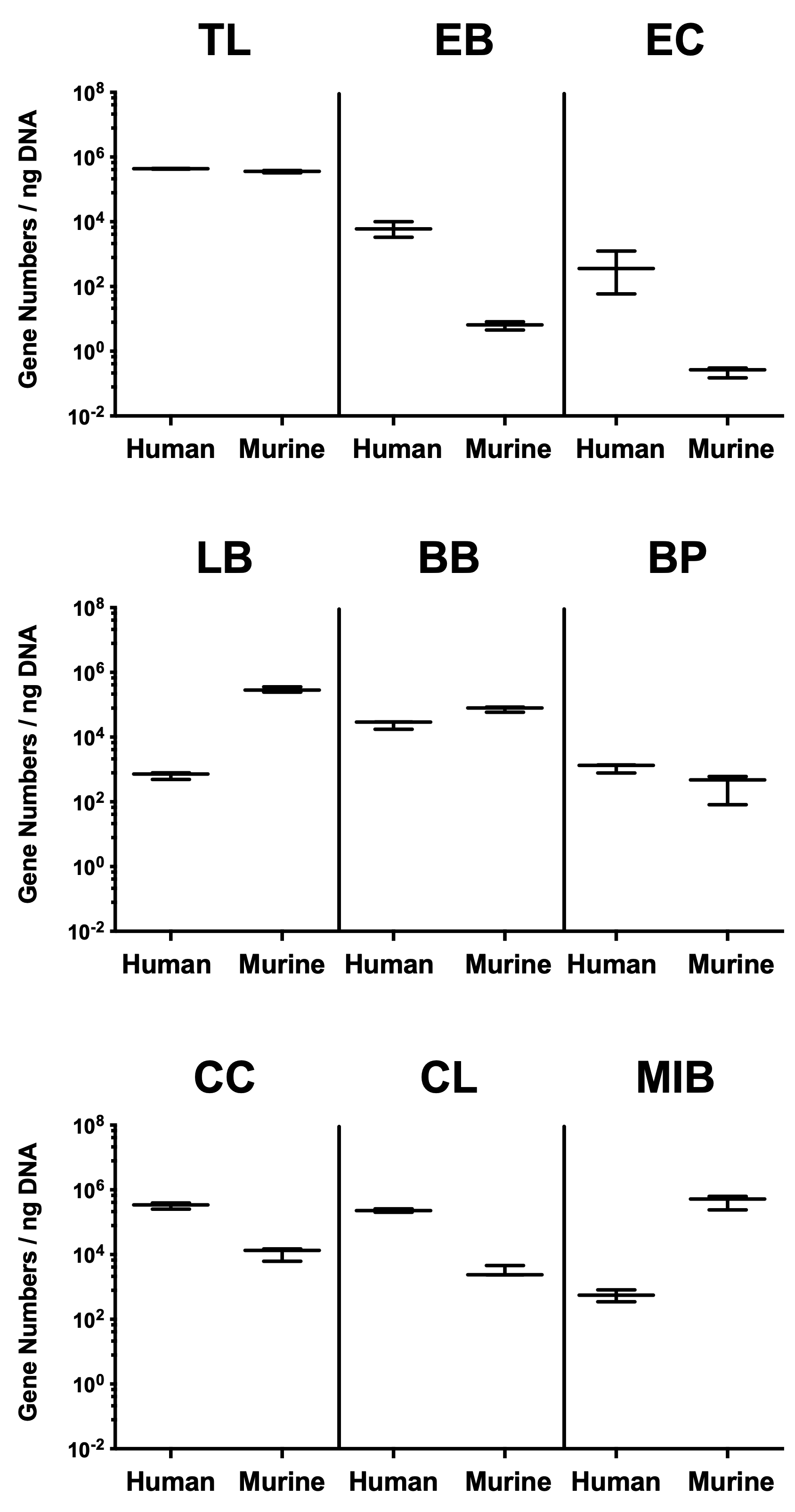

Supplement: Figure S1 — Microbiota composition of suspensions derived from human and murine donors used for fecal microbiota transplantation. Immediately before fecal microbiota transplantation from human and murine donors, the commensal microbiota composition was assessed in respective fecal suspensions (n = 3 each) applying culture-independent 16S rRNA methods quantitating the total eubacterial load (TL) and main bacterial groups including enterobacteria (EB), enterococci (EC), lactobacilli (LB), bifidobacteria (BB), Bacteroides/Prevotella species (BP), Clostridium coccoides group (CC), Clostridium leptum group (CL), and Mouse Intestinal Bacteroides (MIB) and expressed as gene numbers per ng DNA. Box plots represent the 75th and 25th percentiles of medians (black bar inside the boxes) and the total range is indicated. Data were pooled from three independent suspensions. [file Image_1.TIFF]
