# Supplementary material for: Murine Fecal Microbiota Transplantation Alleviates Intestinal and Systemic Immune Responses in Campylobacter jejuni Infected Mice Harboring a Human Gut Microbiota
Source: Front Immunol. 2019 Sep 24;10:2272. doi: 10.3389/fimmu.2019.02272 (PMC6768980; doi:10.3389/fimmu.2019.02272)
Supplement: Figure S2 — Representative photomicrographs illustrating colonic apoptotic epithelial and immune cell responses following murine fecal microbiota transplantation in C. jejuni infected mice harboring a human gut microbiota. Mice with a human gut microbiota were perorally infected with C. jejuni on day (d) 0 and d1 and subjected to murine fecal microbiota transplantation (mFMT) on d7, d8, and d9 post-infection (p.i.) or remained untreated (mock). Photomicrographs representative for four independent experiments illustrate the average numbers of (A) apoptotic epithelial cells (caspase3+), (B) macrophages and monocytes (F4/80+), and (C) T lymphocytes (CD3+), in at least six high power fields (HPF) as quantitatively assessed in colonic paraffin sections applying in situ immunohistochemistry at day 14 p.i. (100x magnification, scale bar 100 μm). [file Image_2.pdf]

A Apoptotic Cells (COLON)

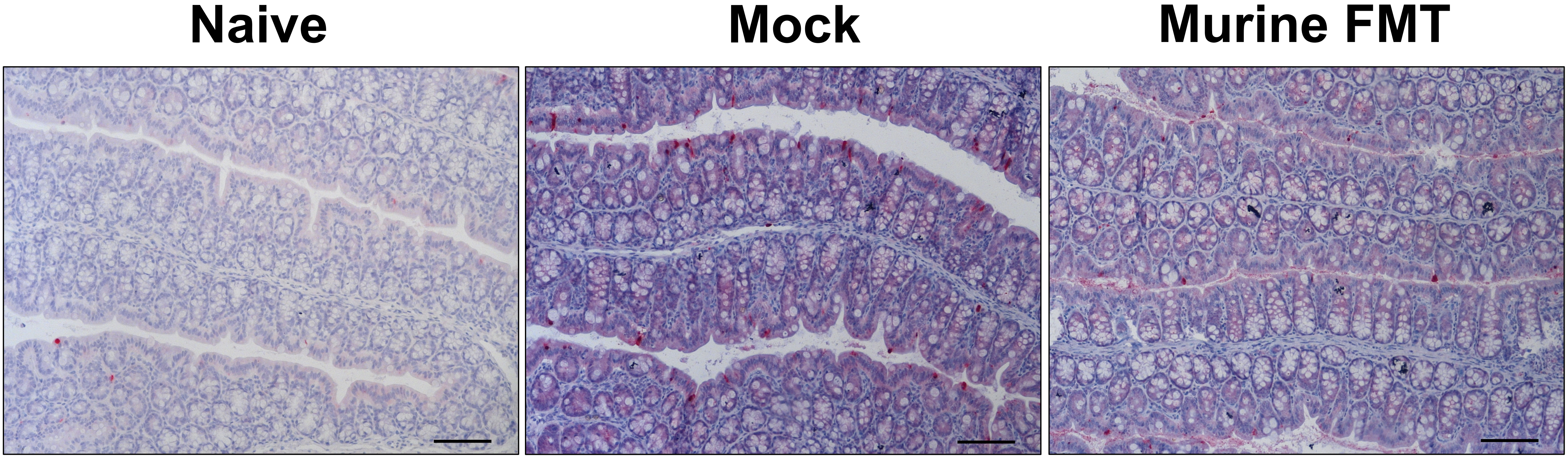

(100 x magnification, scale bar 100 μm)

B

Macrophages / Monocytes (COLON)

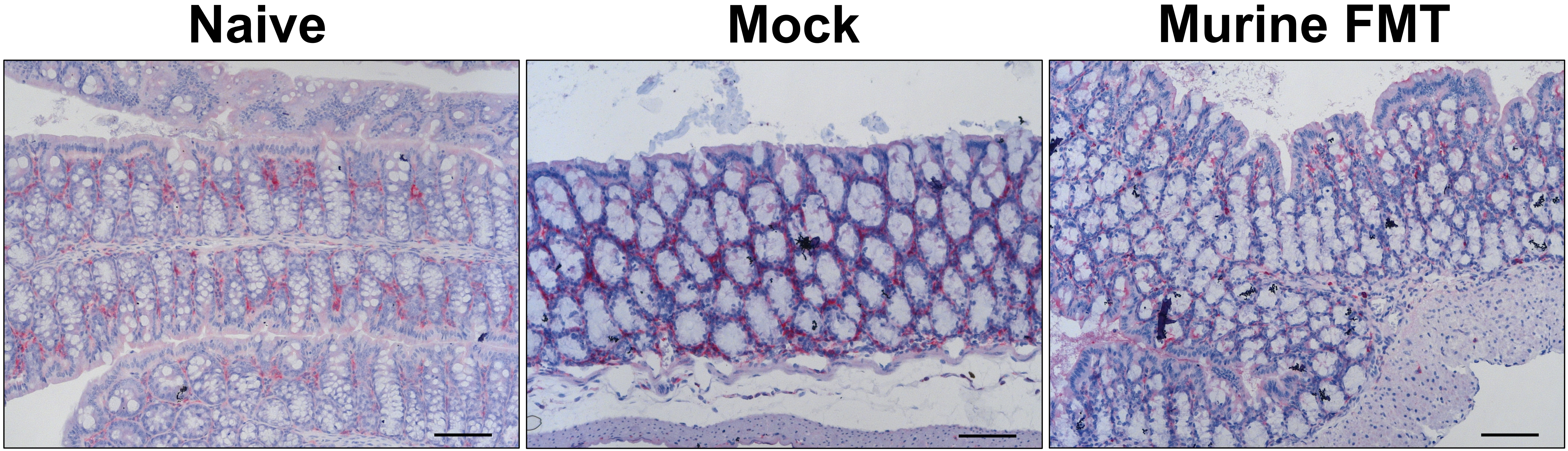

(100 x magnification, scale bar 100 µm)

C

T Lymphocytes (COLON)

Naive | Mock | Murine FMT

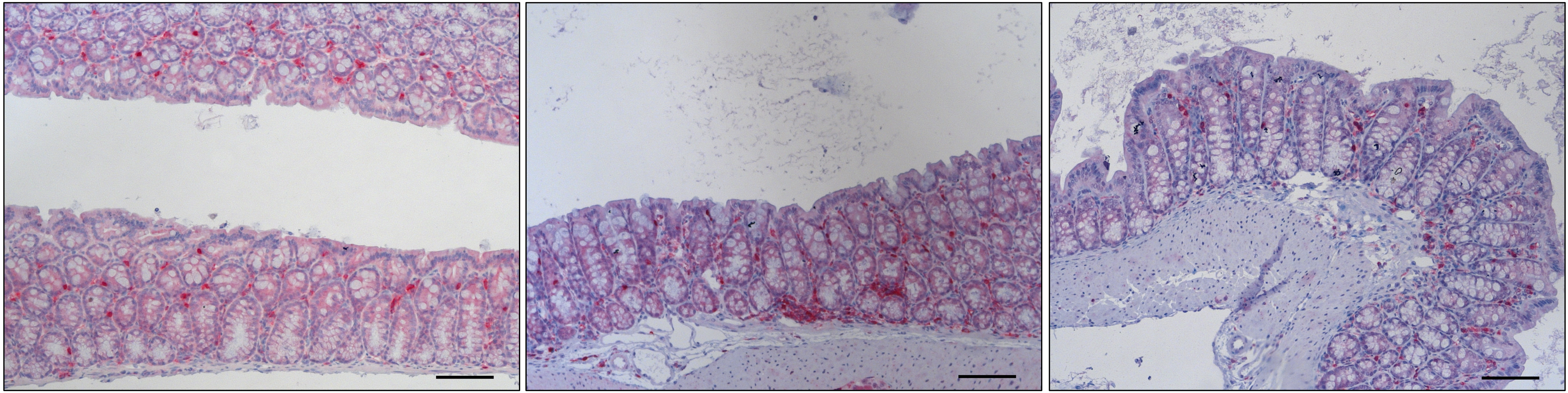

(100 x magnification, scale bar 100 µm)
